# Modeling paraquat-induced lung fibrosis in *C. elegans* reveals KRIT1 as a key regulator of collagen gene transcription

**DOI:** 10.18632/aging.202406

**Published:** 2021-01-20

**Authors:** Gongping Deng, Le Li, Yanhong Ouyang

**Affiliations:** 1 Department of Emergency, Hainan General Hospital, Hainan Affiliated Hospital of Hainan Medical University, Haikou 570311, Hainan, China; 2 Hunan Yuantai Biotechnology Co., Ltd, Changsha 410000, Hunan, China

**Keywords:** paraquat poisoning, lung fibrosis, collagen, KRIT1/KRT-1, Nrf2/SKN-1

## Abstract

Paraquat poisoning causes lung fibrosis, which often results in long-term pulmonary dysfunction. Lung fibrosis has been attributed to collagens accumulation, but the underlying regulatory pathway remains unclear. Here we use the genetically tractable *C. elegans* as a model to study collagen gene transcription in response to paraquat. We find that paraquat robustly up-regulates collagen gene transcription, which is dependent on KRI-1, a poorly studied protein homologous to human KRIT1/CCM1. KRI-1 knockdown prevents paraquat from activating the oxidative stress response transcription factor SKN-1/Nrf2, resulting in reduced collagen transcription and increased paraquat sensitivity. Using human lung fibroblasts (MRC-5), we confirm that both KRIT1 and Nrf2 are required for collagen transcription in response to paraquat. Nrf2 hyper-activation by KEAP1 knockdown bypasses KRIT1 to up-regulate collagen transcription. Our findings on the regulation of collagen gene transcription by paraquat could suggest potential strategies to treat pulmonary fibrosis caused by paraquat poisoning.

## INTRODUCTION

Paraquat (methyl viologen dichloride) in various formulations has been widely used as an herbicide to control weed growth in agriculture. Due to its toxicity, paraquat has been strictly regulated in the developed world. However, in developing countries, paraquat remains widely used and is responsible for a huge number of human fatalities [1]. Despite a long history of adverse societal effects, little is known about the cellular and molecular mechanisms leading to multi-organ failure and death [2, 3]. Currently, there is no cure for paraquat poisoning [4, 5]. Large amounts of paraquat ingestion can cause fulminant organ failure and death within hours. At lower concentrations, paraquat usually leads to kidney failure and lung injury within a few days, followed by progressive pulmonary fibrosis, which still causes over 50% mortality. Survivors of paraquat poisoning suffer from long term pulmonary dysfunction due to lung fibrosis [1, 6].

Lung fibrosis contributes to paraquat-induced mortality; thus, targeting lung fibrosis has been proposed as a treatment [7]. Collagen accumulation is a hallmark of lung fibrosis and has been used to grade the clinical severity of paraquat poisoning [8–11]. Collagens are highly abundant proteins encoded by 44 genes [12]. Collagens are translated in the endoplasmic reticulum (ER), modified along the secretory pathway, and finally deposited in the extracellular matrix where they form insoluble fibers of high tensile strength [13, 14]. Collagen fibers are the major components of skin, bone, tendon, cartilage, blood vessels, and teeth [15–17]. Research in the past aiming to understand how paraquat regulates collagens has delivered mixed results. In rats treated with paraquat, collagens have been shown to increase in lung mince [18], consistent with the clinical observations on patients. However, opposite results have also been reported in rats [19]. There are more conflicting results from cultured cell studies. For example, in cultured fibroblasts, collagens could be increased or decreased, depending on different experimental settings [20, 21].

Paraquat is known to produce high levels of intracellular superoxide through inhibiting mitochondrial complex I, which in turn causes cell death through multiple pathways including apoptosis, autophagy, and necrosis [22–24]. In response to high levels of superoxide, cells upregulate oxidative stress response (OSR). One major OSR is the nuclear factor erythroid-2 related factor 2 (NRF2)-mediated detoxification pathway. Nrf2 is a cap 'n' collar basic leucine zipper transcription factor upregulating a broad spectrum of gene transcription, many of which function to mitigate the superoxide-induced toxicity [25, 26]. Under normal condition, Nrf2 is targeted by KEAP1 for proteasome-mediated degradation in the cytoplasm. Upon oxidative stress, Nrf2 escapes KEAP1-mediated degradation, accumulates in the nucleus, and drives the transcription of target genes. Activation of Nrf2 has been shown to protect paraquat-induced pulmonary fibrosis [27–30].

How paraquat induces collagen transcription and the roles of collagen in paraquat-induced toxicity remain poorly studied. Here, we took advantage of the simple but genetically tractable model organism *C. elegans* to address these questions. We found that collagens were significantly upregulated by paraquat through a poorly studied regulator, KRI-1. KRI-1 activated SKN-1, the *C. elegans* homolog of mammalian Nrf2, to promote collagen transcription in response to paraquat. Enhanced collagen generation protects *C. elegans* from paraquat toxicity. We further confirmed in human lung fibroblasts the conserved regulation of collagen by KRIT1 (homologous to KRI-1) and Nrf2. Together, our study shows a novel genetic circuit that regulates collagen transcription and paraquat toxicity, which could be important for treatment of lung fibrosis resulting from paraquat poisoning.

## RESULTS

### Paraquat increases collagen gene transcription in a KRI-1 dependent manner

**Figure 1:**
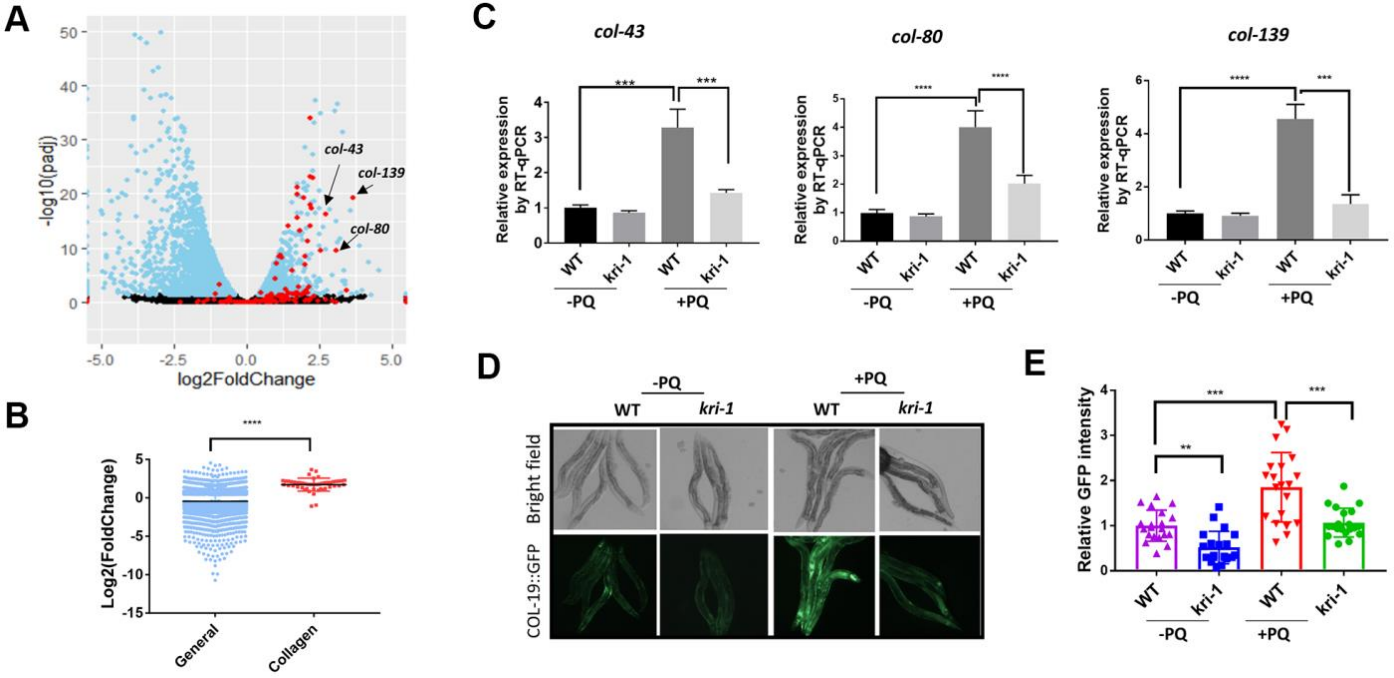
**Paraquat increases collagen gene transcription in a KRI-1 dependent manner.** (**A**) A Volcano Plot showing collagen transcripts were preferentially upregulated after paraquat treatment in *C. elegans*. Dataset GSE123531 was downloaded from NCBI and analyzed by Rstudio. Black indicates transcripts without significantly changed, blue significantly changed and red indicates collagen transcripts. Arrows point to top-ranked transcripts selected for further study. (**B**) Comparison of the average of log2 fold change of collagen genes versus that of all genes (General) significantly changed by paraquat. Error bars indicate the standard deviation. P values were obtained by two tailed, unpaired student’s t-test (****P<0.0001). (**C**) Collagen genes were upregulated by paraquat in a KRI-1-dependent manner. Wild-type (WT) and *kri-1(ok1251)* mutant *C. elegans* were treated with 75 μM paraquat (PQ) from L1 to day-1 of adulthood and total RNA was prepared for RT-qPCR analysis. Shown are the relative expression of *col-43*, *col-80* and *col-139* normalized to non-treated WT control. Error bars indicate the standard deviation of 3 experiments. P values were obtained by two tailed, paired student’s t-test (***P<0.001, ****P<0.0001). (**D**) COL-19::GFP was increased by paraquat in a KRI-1-dependent manner. Wild-type (WT) and *kri-1(ok1251)* mutant *C. elegans* expressing COL-19::GFP fusion proteins were treated with 75 μM paraquat (PQ) from L1 to day-1 of adulthood. Animals were imaged with fluorescence microscope. Shown are representative images from 2 experiments. (**E**) Quantification of GFP intensity in individual worms. 20 worms were selected from 2 independent experiments and quantified through ImageJ software. Data were normalized to the average of WT non-treated control. Error bars indicates the standard deviation of 20 worms. P values were obtained by two tailed, unpaired student’s t-test (**P<0.01, ***P<0.001).

To understand the genetic regulation of collagen accumulation in paraquat-induced lung fibrosis, we took advantage of the genetically tractable *C. elegans* model. By mining RNAseq data (GSE123531) from a previous study [31], we found that collagen genes were generally up-regulated by paraquat (Figure 1A). As shown in the volcano plot, among 181 genes encoding or predicted to encode collagens, 151 were picked up by the RNAseq (red color) and 48 have significant changes. Among 48 collagen genes, 46 were increased and only 2 were decreased by paraquat. Despite the general decrease in global transcription after paraquat treatment, the 48 collagen genes were 1.71 log2 fold changed, or 3.27 fold increased in expression (Figure 1B). COL-43, COL-80, and COL-139 were the top 3 genes affected by paraquat in this study. By examining cluster and protein structure on C. elegans collagens database (CeColDB, http://CeColDB.permalink.cc/.), we find that COL-43, COL-80 and COL-139 belong to different collagen subclasses, e.g. C23a, D07, and B16c, respectively. All contain several Col1-domains and 1 N-Propeptide. COL-139 and COL-43 each contain 1 transmembrane helix while COL-80 contains a Signal Peptide. Since the Col1-domain and N-Propeptide are widely present in many collagens, it seems that paraquat does not preferentially upregulate specific types of collagens.

Next, we asked if KRI-1 was required for collagen gene transcription. KRI-1 was recently shown to be a key mediator of paraquat toxicity in *C. elegans* [32] but its role in collagen transcription has not been reported. By using *kri-1(ok1251)* null mutant animals, we measured the mRNA levels of top-ranked genes from Figure 1A by real-time quantitative PCR (RT-qPCR). We raised WT and *kri-1* mutant worms in 75 μM of paraquat (methyl viologen dichloride hydrate) as determined in [31] from L1 stage to day-1 of adulthood and extracted mRNA from whole worm. The RT-qPCR results showed that lacking KRI-1 largely prevented paraquat from increasing the transcription of *col-43*, *col-80*, and *col-139* (Figure 1C). In addition, by using a GFP-tagged COL-19 fusion protein, we confirmed that COL-19 expression was significantly increased by paraquat and such increase required KRI-1, as *kri-1* null mutant expressing COL-19::GFP was no longer responsive to paraquat treatment (Figure 1D, 1E). As kri-1 could develop slower than control in the presence of paraquat, we also examined COL-19::GFP at day-4 of adulthood. As shown in Supplementary Figure 1, *kri-1* mutants remained much less responsive to PQ than the WT control. These studies identify a novel role of KRI-1 in regulating collagen gene transcription.

### SKN-1 mediates KRI-1 regulation of collagen transcription in response to paraquat treatment

**Figure 2:**
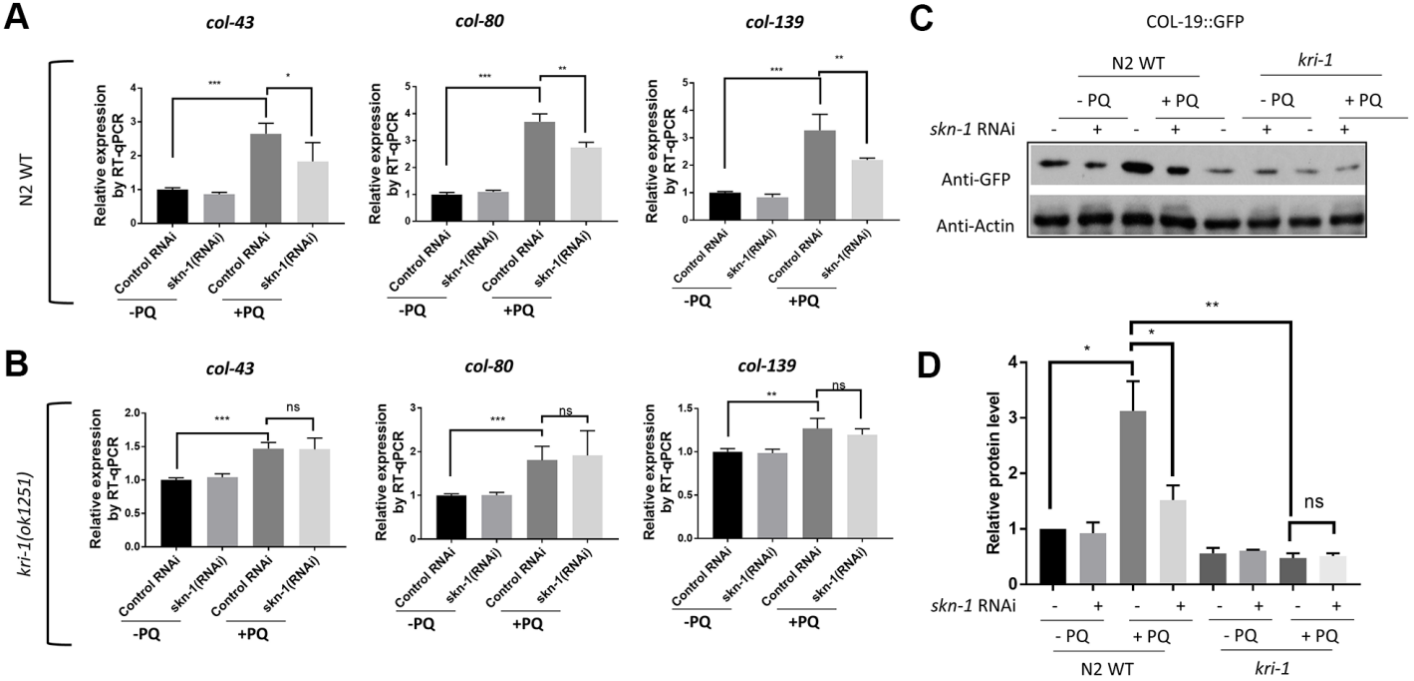
**SKN-1 knockdown inhibits paraquat induction of collagen genes in WT but not *kri-1* mutant worms.** (**A**) Paraquat induction of collagen transcription was impaired by *skn-1* knockdown. WT *C. elegans* were fed control RNAi or *skn-1* RNAi bacteria on agar plates containing 75 μM paraquat (PQ) from L1 to day-1 of adulthood and total RNA was prepared for RT-qPCR analysis. Shown are the relative expression of *col-43*, *col-80* and *col-139* normalized to non-treated controls. Error bars indicate the standard deviation of 3 experiments. P values were obtained by two tailed, paired student’s t-test (*P<0.05, **P<0.01, ***P<0.001). (**B**) *skn-1* knockdown was not additive to *kri-1* mutation in regulating collagen gene transcription. Experiments were performed as in (**A**) except that *kri-1* mutant worms were used. P values were obtained by two tailed, paired student’s t-test (**P<0.01, ***P<0.001, ns, not significant). (**C**) The increase in COL-19::GFP protein levels by paraquat was mitigated by *skn-1* knockdown. *C. elegans* WT and *kri-1* mutant expressing COL-19::GFP were fed control RNAi or *skn-1* RNAi bacteria on agar plates containing 75 μM paraquat (PQ) from L1 to day-1 of adulthood and the total proteins were prepared for Western blot analysis. Actin serves as a loading control. (**D**) Quantification of 3 biological replicates of Western blot data shown in (**C**). Signals on each blot were quantified with ImageJ and normalized to non-treated WT controls. Error bars indicate the standard deviation of 3 biological repeats. P values were obtained by two tailed, paired student’s t-test (*P <0.05, **P<0.01, ns, not significant).

The oxidative stress responsive transcription factor SKN-1 positively regulates collagen transcription to delay aging in *C. elegans* [33]. We wondered if KRI-1 would regulate SKN-1 to promote collagen gene transcription in response to paraquat treatment. First, we knocked down SKN-1 transcription by RNAi and asked if such treatment would block paraquat from inducing collagen transcription. Worms were fed HT115 bacteria expressing double-stranded RNA targeting *skn-1* gene from L1 stage to day-1 of adulthood in the presence or absence of 75 μM paraquat. RT-qPCR demonstrated that the transcription of *col-43*, *col-80* and *col-139* were all reduced by *skn-1* knockdown (Figure 2A). *skn-1* knockdown in *kri-1* mutant did not further decrease the expression of above collagen genes (Figure 2B), suggesting that KRI-1 and SKN-1 function in the same pathway to regulate collagen transcription. By using Western blot to detect GFP levels, we further confirmed the genetic interaction between KRI-1 and SKN-1 on collagen gene transcription; paraquat-induced COL-19:GFP up-regulation was blunted by SKN-1 knockdown and largely blocked by *kri-1* knockout, but was not additively affected by both (Figure 2C, 2D).

**Figure 3:**
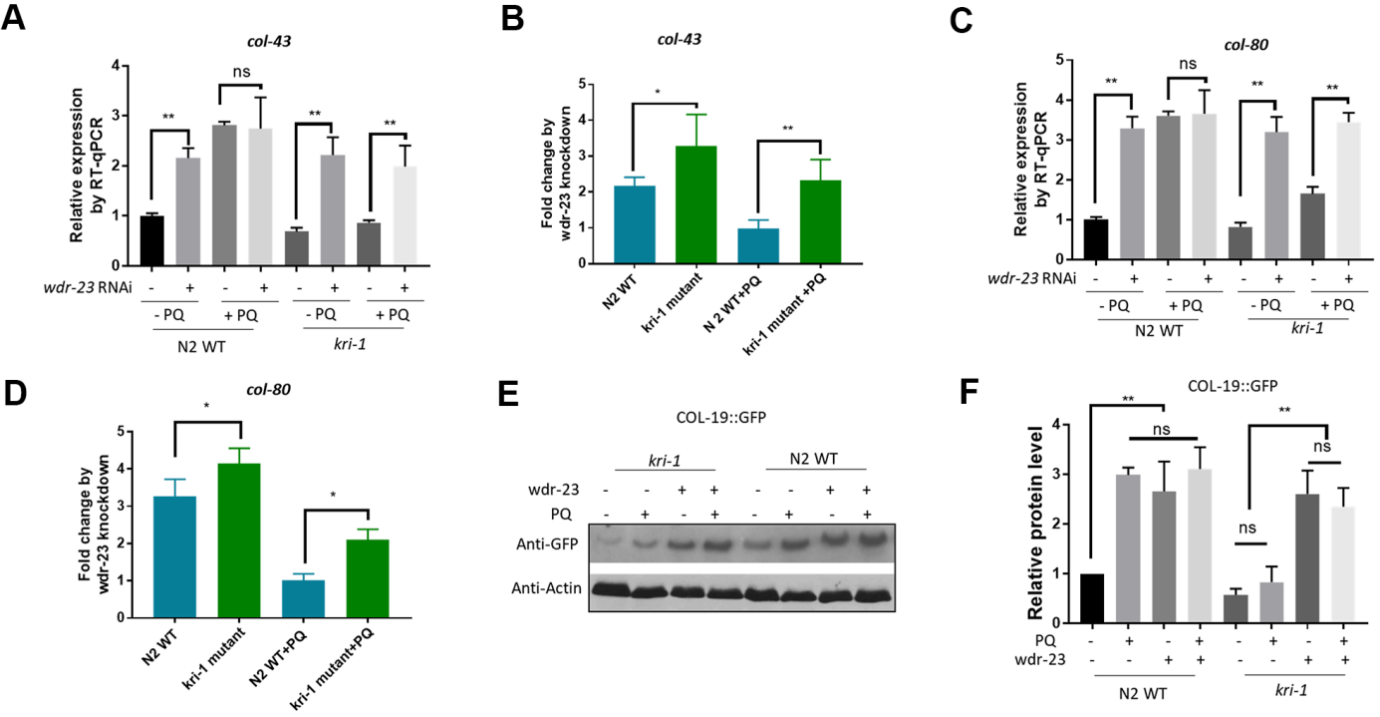
**Hyperactivation of SKN-1 increases collagen gene transcription preferentially in *kri-1* mutant worms.** (**A**) Hyperactivation of SKN-1 by WDR-23 knockdown up-regulated *col-43* transcription. WT *C. elegans* were fed control RNAi or *wdr-23* RNAi bacteria on agar plate containing 75 μM paraquat (PQ) from L1 to day-1 of adulthood and total RNA was prepared for RT-qPCR analysis. Error bars indicate the standard deviation of 3 experiments. P values were obtained by two tailed, paired student’s t-test (**P<0.01, ns, not significant). (**B**) SKN-1 hyperactivation up-regulated *col-43* expression preferentially in *kri-1* mutant. Data from (**A**) were shown in fold changes by *wdr-23* RNAi knockdown. P values were obtained by two tailed, paired student’s t-test (*P<0.05; **P<0.01). (**C**) *wdr-23* knockdown up-regulated the transcription of collagen gene *col-80*. Experiments were conducted as in (**A**) except *col-80* mRNA levels were examined. P values were obtained by two tailed, paired student’s t-test (** <0.01, ns, not significant). (**D**) *wdr-23* knockdown up-regulated *col-80* transcription preferentially in *kri-1* mutant. Data in (**C**) were shown in fold change by *wdr-23* RNAi knockdown. P values were obtained by two tailed, paired student’s t-test (*P<0.05). (**E**) *wdr-23* knockdown partially rescued the collagen transcription defect in *kri-1* mutant. *C. elegans* WT and *kri-1* mutant expressing COL-19::GFP were fed control RNAi or *wdr-23* RNAi bacteria on agar plate containing 75 μM paraquat (PQ) from L1 to day-1 of adulthood and the total proteins were prepared for Western blot analysis. Actin serves as a loading control. (**F**) Quantification of 3 biological replicates of Western blot data as shown in (**E**). Signals on each blot were quantified with ImageJ and normalized to non-treated WT controls. Error bars indicate the standard deviation of 3 biological repeats. P values were obtained by two tailed, paired student’s t-test (**P<0.01, ns, not significant).

We next asked if SKN-1 hyperactivation could be epistatic to *kri-1* mutant. WDR-23 is homologous to human KEAP1, a negative regulator of Nrf2; knocking down *wdr-23* expression constitutively activates SKN-1 in *C. elegans* [34]. We knocked down *wdr-23* from L1 in the presence and absence of 75 μM paraquat. Indeed, *wdr-23* knockdown significantly increased the transcription of *col-43* and *col-80* in both WT and *kri-1* mutant worms (Figure 3A, 3C). The normalized fold changes were higher in *kri-1* mutant (Figure 3B, 3D) as compared to N2 WT, suggesting that Nrf2 are epistatic to KRI-1 in collagen transcription. Further supporting this result, western blot analysis showed that *wdr-23* knockdown increased COL-19::GFP levels regardless of paraquat treatment in *kri-1* mutant (Figure 3E, 3F), suggesting that KRI-1 regulates collagen transcription at least partly through SKN-1.

### Collagen up-regulation by SKN-1 is required for protection from oxidative stress

**Figure 4:**
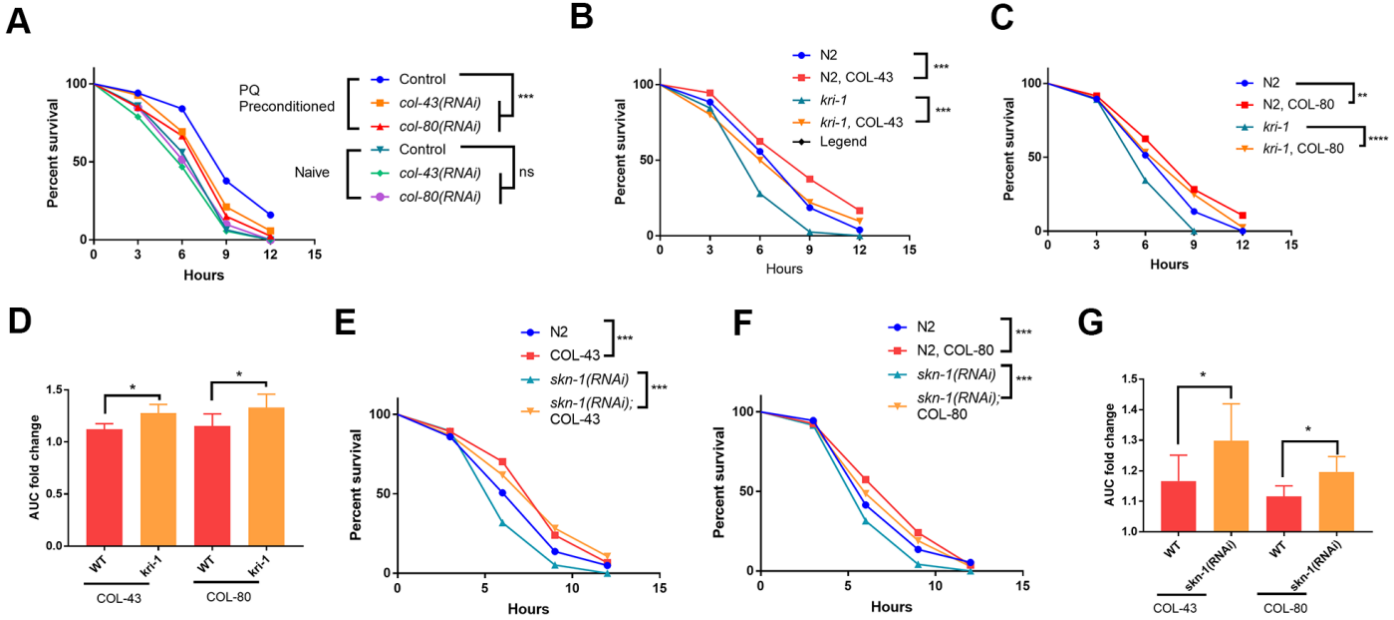
**Collagen up-regulation by SKN-1 is required for protection from paraquat toxicity.** (**A**) Low paraquat (PQ) preconditioning increased the tolerance to high PQ toxicity in a collagen-dependent manner. WT *C. elegans* were fed control RNAi, *col-43 or col-80* RNAi bacteria on agar plate containing 75 μM paraquat (PQ) from L1 to day-1 of adulthood. Animals (n>100 each for 3 experiments) were transferred to agar plate containing 200 mM paraquat and examined every 3 hours for 12 hours. Survival data were statistically analyzed by log-rank test (ns, not significant, ****P<0.0001). (**B**, **C**) Collagen COL-43 and COL-80 overexpression increased the tolerance to paraquat toxicity in both WT and *kri-1* mutant worms. Young adult worms (n>100 each for 3 experiments) expressing collagen COL-43 and COL-80 were subjected to paraquat toxicity assay as shown in (**A**). Survival data were statistically analyzed by log-rank test (***P<0.001). (**D**) Collagen COL-43 and COL-80 overexpression preferentially increased the tolerance to paraquat toxicity in *kri-1* mutant worms. Area under the curve (AUC) in (**B**) and (**C**) was shown for 3 biological replicates and the fold changes were statistically analyzed by using paired student’s t-test (*P<0.05). (**E**, **F**) Collagen COL-43 and COL-80 overexpression increased the tolerance to paraquat toxicity in both WT and *skn-1(RNAi)* worms. WT worms (n>100 each for 3 experiments) expressing collagen COL-43 or COL-80 were fed *skn-1* RNAi bacteria from L1 stage to young adult stage and subjected to paraquat toxicity assay as shown in (**A**). Survival data were statistically analyzed by log-rank test (***P<0.001). (**G**) Collagen COL-43 and COL-80 overexpression increased the tolerance to paraquat toxicity preferentially in *skn-1(RNAi)* worms. Area under the curve (AUC) in (**E**) and (**F**) was shown for 3 biological replicates and the fold changes were statistically analyzed by using paired student’s t-test (*P<0.05).

Since collagen has been shown to improve health and extend lifespan in *C. elegans* [33], the transcriptional induction of collagen gene upon paraquat exposure could function as a protective mechanism against oxidative stress from paraquat. To test this, we first examined if loss of collagen could render worms sensitive to paraquat. Although there are 181 collagen genes in *C. elegans*, knocking down a single collagen gene has been shown to compromise the expression of other collagen genes [33]. We knocked down *col-43* and *col-80* individually and raised worms in the presence or absence of 75 μM paraquat. At day-1 of adulthood, we tested their tolerance to oxidative stress by challenging worms with 200 mM paraquat for 8 hours [35]. As shown in toxicity assay in Figure 4A, worms preconditioned with 75 μM paraquat tolerated much better than non-treated controls. However, knocking down either *col-43* or *col-80* significantly compromised the acquired tolerance to paraquat toxicity (Figure 4A).

Next, we tested if collagen overexpression would rescue the sensitive phenotype of *kri-1* and *skn-1* mutants to paraquat. Interestingly, although there are many collagen genes, overexpressing single collagen gene has been shown to promote longevity in *C. elegans* [33]. By using transgenic worms expressing high copy number of extrachromosomal collagen genes (Supplementary Figure 2), we showed that COL-43 and COL-80 overexpression significantly increased the resistance to paraquat toxicity in all WT *kri-1* mutant and *skn-1(RNAi)* animals (Figure 4B, 4C, 4E, 4F). Consistent with a previous study [32], *kri-1* mutant and *skn-1(RNAi)* animals were sensitive to paraquat, but such sensitivity was partly rescued by COL-43 and COL-80 overexpression. By measuring the area under the curve (AUC) and calculated the fold increase, we showed that both collagen overexpression preferentially increased paraquat tolerance in *kri-1* and *skn-1(RNAi)* animals as compared to WT (Figure 4D, 4G), further confirming the specific roles of KRI-1 and SKN-1 in the regulation of collagen transcription.

We were also interested in knowing if promoting resistance to paraquat could result in extended lifespan. Overexpressing several collagen genes such as COL-10, COL-13 and COL-120 have been shown to extend lifespan [33]. Consistently, we found that COL-43 and COL-80 overexpression also extended lifespan of WT animals (Supplementary Figure 3B).

### Conserved roles of Krit1 and Nrf2 in collagen transcription in human lung fibroblasts

**Figure 5:**
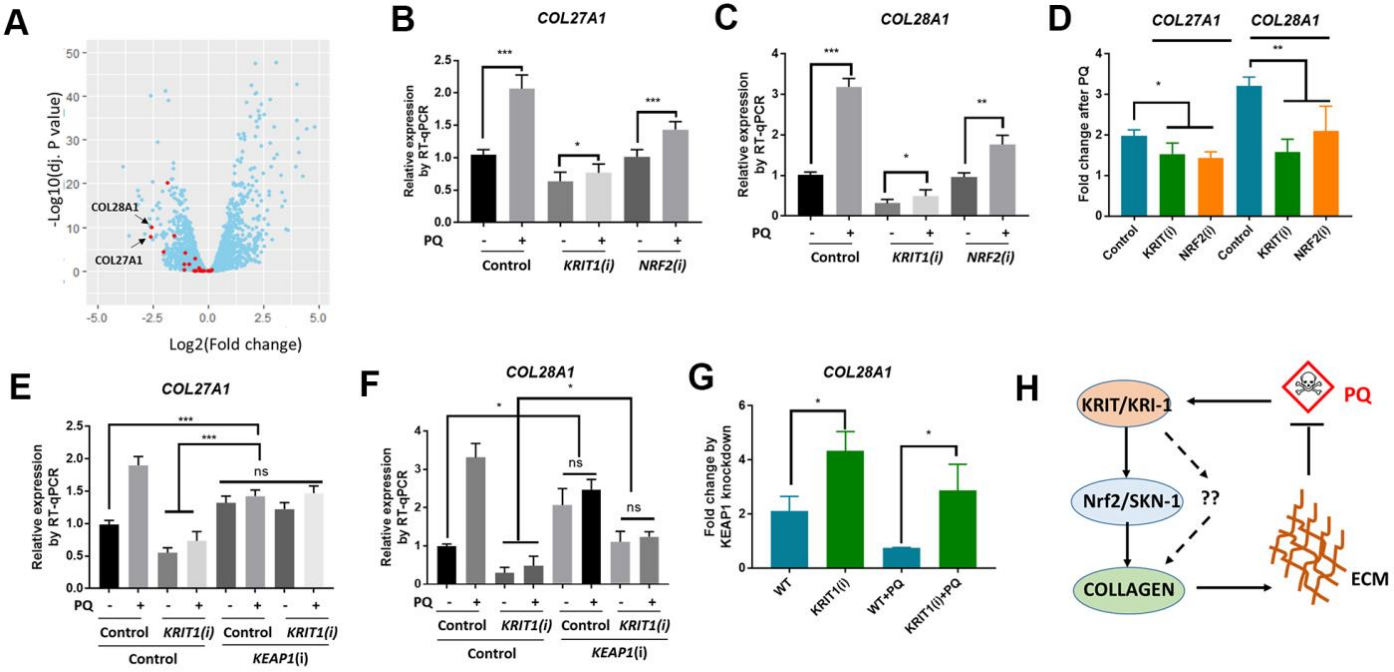
**Conserved roles of Krit1 and Nrf2 in collagen transcription in human lung fibroblasts.** (**A**) A Volcano Plot showing collagen genes expression were down-regulated in krit1-knockdown mouse primary brain microvascular endothelial cells. Dataset GSE85657 was downloaded from NCBI and analyzed by Rstudio. Red data points indicate collagen transcripts and blue other transcripts detected by RNA sequencing. Arrows point to top-ranked transcripts selected for further study. (**B**, **C**) Paraquat up-regulated *COL27A1* and *COL28A1* expression in human lung fibroblasts. MRC-5 cells were transfected with *KRIT1* and *NRF2* siRNAs and treated with 300 μM of paraquat for 48 hours. Total mRNA was purified and reverse transcribed for RT-qPCR analysis. Error bars indicate the standard deviation of 3 experiments. P values were obtained by two tailed, paired student’s t-test (*P<0.05, **P<0.01, ***P<0.001). (**D**) *KRIT1* and *NRF2* knockdown inhibit paraquat induction of collagen transcription. Data from (**B**, **C**) were transformed to fold changes by paraquat treatment and statistically tested by two tailed, paired student’s t-test (*P<0.05, **P<0.01). (**F**) *KEAP1* knockdown up-regulated collagen transcription in human lung fibroblasts. Experiments were conducted as in (**B**, **C**) except that *KEAP1* siRNA was used. Error bars indicate the standard deviation of 3 experiments. P values were obtained by two tailed, paired student’s t-test (*P<0.05, ***P<0.001). (**G**) *KEAP1* knockdown up-regulated collagen transcription preferentially in *KRIT1*-knockdown cells. Data from (**D**, **E**) were transformed into fold change by *KEAP1* knockdown and statistically tested by two tailed, paired student’s t-test (*P<0.05). (**H**) A working model showing KRIT1/KRI-1 regulation of collagen transcription. Paraquat treatment could generate protective signals through KRIT1/KRI-1, which in turn activates Nrf2/SKN-1 pathway to promote collagen transcription. Up-regulation of collagen may serve to protect cells and organisms from paraquat toxicity.

We were interested to know if our findings in *C. elegans* would be conserved in human cells. In *C. elegans*, we observed that even without paraquat treatment, *kri-1* mutation had already reduced collagen gene expression (Figure 1D, 2C), suggesting that *kri-1* is also required for the basal collagen expression under normal condition. This prompted us to investigate into the public GEO dataset deposited at National Center for Biotechnology Information (NCBI). The GSE85657 contains RNAseq data of KRIT1 (human homolog of KRI-1) knockdown in mouse primary brain microvascular endothelial cells [36]. We took advantage of this resource and did a Volcano Plot of 11653 transcripts including 26 collagen genes. We found that in the KRIT1-depleted cells, 22 out of 26 collagen genes were decreased as compared to those in WT control (Figure 5A, red). The mean expression of all the 26 collagen genes was -0.71 in log2 fold change, which equals to 0.61 fold change as compared to those in WT control. These results support our findings in *C. elegans*, suggesting that KRIT1 could function in a conserved manner in human cells.

In human lung fibroblast cell line MRC-5, paraquat has been shown to increase collagen transcription [21]. We asked if KRIT1 and Nrf2 (human homolog of SKN-1) would be required for paraquat to increase collagen gene transcription. First, we knocked down KRIT1 and NRF2 individually, then treated MRC-5 cells with 300 μM paraquat for 48 hours as determined by the previous paper [21]. We extracted mRNA for RT-qPCR analysis of 2 top-ranked collagen genes *COL28A1* and *COL27A1* in Figure 5A (Arrows). COL28A1 and COL27A1 belong to their distinct collagen types, XXVIII and XXVII, respectively. COL28A1 belongs to a class of collagens containing von Willebrand factor type A (VWFA) domains and could be involved in collagen chain trimerization and degradation of the extracellular matrix [37]. COL27A1 is related to the "fibrillar" class of collagens and may play a role in development of the skeleton [38, 39]. Interestingly, they have been predicted to be functional partners based on STRING, the protein-protein interaction network. Consistent with our study in *C. elegans*, *COL28A1* and *COL27A1* expression were elevated after paraquat treatment in MRC-5 cells (Figure 5B, 5C). Interestingly, *KRIT1* knockdown reduced basal expression of both *COL28A1* and *COL27A1,* consistent with the RNAseq data shown in Figure 1A. Although paraquat still increased collagen gene expression in *KRIT1-* and *NRF2-*knockdown cells, the fold change was significantly reduced (Figure 5D), suggesting specific roles of KRIT1 and Nrf2 in paraquat toxicity.

We further tested if Nrf2 functions downstream of KRIT1 for collagen transcription. Similar to the study in *C. elegans*, we activated Nrf2 by knocking down its negative regulator KEAP1 and examined *COL27A1* and *COL28A1* mRNA expression. Consistently, *KEAP1* knockdown upregulated *COL27A1* mRNA levels in both WT and *KRIT1*-knockdown MRC-5 cells, rescuing the low expression in *KRIT1*-knockdown cells to WT levels (Figure 5E). A similar pattern was observed for *COL28A1* except that the low expression in *KRIT1*-knockdown cells was not fully rescued (Figure 5F). However, after comparing the fold change, we found that KEAP1 siRNA knockdown had stronger effect on *COL28A1* expression in *KRIT1*-knockdown cells than control cells (Figure 5G), suggesting KRIT1 regulates collagen at least partly through Nrf2 pathway. Interestingly, for unknown reasons, KEAP1 knockdown in paraquat-treated cells reduced the expression of both collagen genes (Figure 5E, 5F).

## DISCUSSION

We have shown several novel experimental results in this study. First, we implicated KRIT1/KRI-1 in collagen transcription in both *C. elegans* and MRC-5 human lung fibroblasts. Second, we established the interaction between Nrf2/SKN-1 and KRIT1/KRI-1 in the regulation of collagen transcription. Third, we showed that increased collagen serves to protect *C. elegans* from paraquat toxicity. These novel findings together suggest a stress responsive pathway where paraquat generates mitochondrial stress signals, transmits through KRIT1/KRI-1 and Nrf2/SKN-1, and activates nuclear transcription of collagen genes, leading to enhanced extracellular matrix (ECM) that serves to protect cells and tissue from paraquat toxicity (Figure 5H).

Our results were supported by global gene transcription studies from other research groups. By mining public RNA sequence data [31], we find that paraquat preferentially upregulates collagen gene transcription despite the global decrease in genome-wide transcription in *C. elegans* (Figure 1A). Similarly, our discovery of the negative role of KRIT1 in collagen transcription is supported by public RNAseq data in mouse primary brain microvascular endothelial cells (Figure 5A) [36]. Interestingly, the fold change of collagen gene transcription is generally much higher in the two RNAseq data as compared to our RT-qPCR results. This could be simply resulted from variations introduced by different methods of sample preparation and different sensitivity of detecting technologies. Nevertheless, the rapid accumulation of the publicly available data has provided valuable resources to compare experimental results across different labs, leading to more reliable data that help better understand the biology of paraquat toxicity.

The application of the simple model organism *C. elegans* has rapidly delivered informative experimental results on paraquat toxicity. By sequence, *C. elegans* roughly has about 83% of proteins with human homologs and ~40% matching known human transcripts [40, 41]. As in our study, all KRI-1/KRIT1, SKN-1/Nrf2 and WDR-23/KEAP1 are highly conserved in either the primary sequence or function or both [32, 34, 42]. To test if our findings in *C. elegans* are conserved in human cells, we used human lung fibroblast (MRC-5 cells) and confirmed that indeed, the regulation of collagen by KRIT1 and NRF2 is conserved. However, *in vitro* cell models lack extracellular matrices, so functional study of collagen in paraquat toxicity is largely not possible. Therefore, whether collagen accumulation in response to paraquat could protect mammalian cells from oxidative stress as shown in the *C. elegans* model needs considerable amount of work in mice or rats.

The detailed mechanisms of collagen transcriptional regulation by Nrf2/SKN-1 remain unclear. Although Nrf2 is a DNA-binding transcriptional factor, the direct regulation of collagen genes by Nrf2 has not been reported. In this study, the promoters of collagen genes we examined (*col-43*, *col-80*, and *col-139* *in C. elegans* and COL28A1 and COL27A1 in mice) do not contain conserved Nrf2 binding sites, consistent with a previous study [33]. Therefore, we prefer a model whereby Nrf2 activates other transcription factors to indirectly promote collagen transcription. How KRI-1/KRIT1 regulates Nrf2 is also unknown. KRI-1/KRIT1 has been reported to mediate ROS metabolism [32, 43, 44]. Since upstream regulators of Nrf2 including KEAP1 have been shown to be directly modified by superoxide [45, 46], it is possible that KRI-1/KRIT1 regulates Nrf2 through superoxide or other reactive oxygen species (ROS). Further studies will be needed to fully understand the mechanisms in the upstream and downstream of KRIT1, Nrf2 that modulate collagen transcription.

How increased collagen contributes to paraquat (PQ) resistance and lifespan extension remains unclear. An easy explanation is that more collagens could simply increase the cuticle barrier so as to reduce the permeability of PQ. However, by testing the cuticle permeability of Hoechst staining, we show that it is not likely that overexpressing COL-43 or COL-80 could improve the cuticle barrier (Supplementary Figure 3A). This is consistent with the overexpression of COL-19, where COL-19 seems to be accumulated in the hypodermis rather than improving cuticles (Figure 1D–1F). Therefore, alternative mechanisms should be tested in the future. For example, collagen expression could modulate the signaling pathways at the cell membrane in hypodermis, leading to dampened antioxidant defense in other tissues.

## MATERIALS AND METHODS

### *C. elegans* maintenance and RNAi knockdown

Animals were maintained at 25° C on nematode growth medium (NGM) agar plates seeded with OP-50 bacteria. NGM agar plates were prepared as described before [47]. Information regarding N2 Bristol wild-type, *kri-1(ok1251)* mutant and strains expressing COL-19::GFP, COL-43 and COL-80 were detailed in the Supplemental Information section. RNAi clones were originally from RNAi library constructed by the Ahringer group [48]. RNAi knockdown was achieved by feeding worms HT115 bacteria expressing double-stranded RNA (dsRNA) of target genes on NGM agar plates. Specifically, RNAi bacteria were cultured to log phase in LB liquid medium containing 50 ug/mL carbenicillin then seeded on NGM agar plate containing 50 ug/mL carbenicillin and 1 mM isopropyl β-D-1-thiogalactopyranoside (IPTG) for 2 days. L1 stage worms were transferred to and maintained on the RNAi plate until day-1 of adulthood.

### MRC-5 cell culture and siRNA knockdown

MRC-5 cells were originally obtained from ATCC and maintained in Dulbecco’s minimal essential medium (DMEM) supplemented with 10% heat-inactivated fetal calf serum (FCS) at 37° C in 5% CO2 humidified incubator. Fibroblasts between passages 25 and 35 were used for all experiments. For siRNA knockdown, cells at 60% confluence were transiently transfected with siRNA by using lipofectamine 2000 (ThermoFisher Scientific). After 24 hours, cells were treated with paraquat at the final concentration of 300 μM for 48 hours. The siRNA information for KRIT1, KEAP1 and NRF2 is shown in Supplementary Table 1.

### Real time quantitative PCR (RT-qPCR)

*C. elegans* day-1 adult animals were homogenized by sonication and total RNA was extracted by TRIZOL reagent. Total RNA from MRC-5 cells was obtained by purification from cell monolayers using the PureLink RNA Mini Kit. Total RNA was treated with DNase I to remove DNA contaminants, then reverse transcribed to cDNA with High-Capacity cDNA reverse transcription kit (Invitrogen) according to the manufacturer's instruction. RT-qPCR was conducted in SYBR Green PCR Master Mix (Applied Biosystems) in triplicate using the ABI Prism 7300 Sequence Detection System. Actin gene (*act-1*) was used as internal control for *C. elegans* and *GAPDH* for human cells. Primers sets were designed by ProbeFinder software (version 2.45) of the Universal Probe Library from Roche. The primer sets are listed in Supplementary Table 2.

### Western blot

*C. elegans* day-1 adult animals were homogenized by sonication in RIPA Lysis and Extraction Buffer supplemented with Halt™ Protease Inhibitor Cocktail (ThermoFisher Scientific). The protein concentration was measured by Pierce™ BCA Protein Assay Kit and 100 μg of the total proteins for each sample was denatured by heating in SDS-loading buffer at 90° C for 5 min. 25ug of the total proteins were separated on SDS-PAGE and transferred to PVDF membrane. Western blot was carried out by blocking the membrane in 5% non-fat milk in PBS for 30 min, incubating with mouse anti-GFP or mouse anti-β-actin primary antibody (Promab Biotechnologies #20144, #20270) for 1 hour, washing extensively, and then finally incubating with an anti-mouse, HRP-conjugated secondary antibody for 30 min. HRP signal were detected with LumiGLO® reagent autoradiographed with X-ray film.

### Paraquat toxicity assay

Paraquat toxicity assay in *C. elegans* is described in detail in [35]. Briefly, synchronized worms were raised to young adult stage and transferred to NGM agar plate containing 200 mM paraquat. 5 plates containing roughly 40 worms/plate were assayed each time and experiments were repeated 3 times. Live and dead worms were recorded every 3 hours until 12 hours. Death is defined by worms that were no longer moving when touched by a platinum tip. Percentage Survival were calculated and plotted with GraphPad Prism software.

### Data analysis, visualization and statistics

RNAseq Datasets of GSE123531 and GSE85657 were downloaded from NCBI website and analyzed by using Rstudio software loaded with ggplot2 package. Volcano Plots were drawn in Rstudio using total data points and data points for collagen genes (red). COL-19::GFP signal and western blot signal were quantified in ImageJ software. Bar graph were generated using GraphPad Prism software. Statistical analysis methods were indicated in individual figure legends, which were also generated using GraphPad Prism software.

## Supplementary Material

Supplementary Information

Supplementary Figures

Supplementary Tables
