# Supplementary material for: Modeling paraquat-induced lung fibrosis in C. elegans reveals KRIT1 as a key regulator of collagen gene transcription
Source: Aging (Albany NY). 2021 Jan 20;13(3):4452–67. doi: 10.18632/aging.202406 (PMC7906160; doi:10.18632/aging.202406)
Supplement: Supplementary Information [file aging-13-202406-s001.pdf]

SUPPLEMENTARY INFORMATION

C. elegan strains

The wild-type strains used in this study was N2 Bristol wild-type, to which all other mutant strains and transgenic strains were crossed at least 3 times to remove background mutation. N2 (Bristol) and *kri-1(ok1251)* (CF2052) mutant and COL-19::GFP (TP12) were obtained from CAENORHABDITIS GENETICS CENTER (CGC).

Transgenic *C. elegan* strains

To generate COL-43 and COL-80 overexpressing lines, genomic DNA sequence flanked by 500bp in the upstream and downstream were amplified by PCR. PCR products were purified through QIAgene PCR purification kit and inject with *rol-6* co-injection marker into the germline of young adult worms. Individual roller offspring was picked and maintained to get stable lines. 3 lines were for each were examined for COL-43 and COL-80 expression by RT-qPCR and the strains with highest expression were used in this study (Supplementary Figure 1).

Hoechst 33342 staining

Day-1 adult worms were stained in M9 buffer containing 1 µg/ml Hoechst 33342 at room temperature for 15 min, then washed extensively with M9 buffer. Nuclei stained with Hoechst in the tail of an animal were counted under fluorescent microscope. Animals were classified into 4 groups based on how many nuclei were stained in the tail: high (>10 nuclei stained), medium (6-10 nuclei stained), low (1-5 nuclei stained) and no Hoechst staining.

Lifespan assay

For lifespan assay in C. elegans, age-matched L1 worms were raised on either OP-50 or RNAi bacteria plate until L4/young adult stage, then 50 µM FUDR (5-Fluoro-2′-deoxyuridine) was added to inhibit reproduction. Worms may be transferred to new plates as required. The number of live and dead worms was recorded every 2 or 3 days starting from day 10. Exploded and bagged worms and worms with protruding vulva were censored. Death was defined as lack of any visible movement for 5 seconds after touching the tail and head with a platinum wire.

REFERENCES

1. Singh A, Boldin-Adamsky S, Thimmulappa RK, Rath SK, Ashush H, Coulter J, Blackford A, Goodman SN, Bunz F, Watson WH, Gabrielson E, Feinstein E, Biswal S. RNAi-mediated silencing of nuclear factor erythroid-2-related factor 2 gene expression in non-small cell lung cancer inhibits tumor growth and increases efficacy of chemotherapy. Cancer Res. 2008; 68:7975–84. https://doi.org/10.1158/0008-5472.CAN-08-1401 PMID:18829555

2. Devling TW, Lindsay CD, McLellan LI, McMahon M, Hayes JD. Utility of siRNA against Keap1 as a strategy to stimulate a cancer chemopreventive phenotype. Proc Natl Acad Sci USA. 2005; 102:7280–85A. https://doi.org/10.1073/pnas.0501475102 PMID:15883370

3. Goitre L, De Luca E, Braggion S, Trapani E, Guglielmotto M, Biasi F, Forni M, Moglia A, Trabalzini L, Retta SF. KRIT1 loss of function causes a ROS-dependent upregulation of c-Jun. Free Radic Biol Med. 2014; 68:134–47. https://doi.org/10.1016/j.freeradbiomed.2013.11.020 PMID:24291398

4. Serobyan V, Kontarakis Z, El-Brolosy MA, Welker JM, Tolstenkov O, Saadeldein AM, Retzer N, Gottschalk A, Wehman AM, Stainier DY. Transcriptional adaptation in Caenorhabditis elegans. Elife. 2020; 9:e50014. https://doi.org/10.7554/eLife.50014 PMID:31951195

5. Ham S, Harrison C, Southwick G, Temple-Smith P. Selection of internal control genes for analysis of gene expression in normal and diseased human dermal fibroblasts using quantitative real-time PCR. Exp Dermatol. 2016; 25:911–14. https://doi.org/10.1111/exd.13091 PMID:27245982
