# Supplementary material for: Modeling paraquat-induced lung fibrosis in C. elegans reveals KRIT1 as a key regulator of collagen gene transcription
Source: Aging (Albany NY). 2021 Jan 20;13(3):4452–67. doi: 10.18632/aging.202406 (PMC7906160; doi:10.18632/aging.202406)
Supplement: Supplementary Figures [file aging-13-202406-s002.pdf]

SUPPLEMENTARY FIGURES

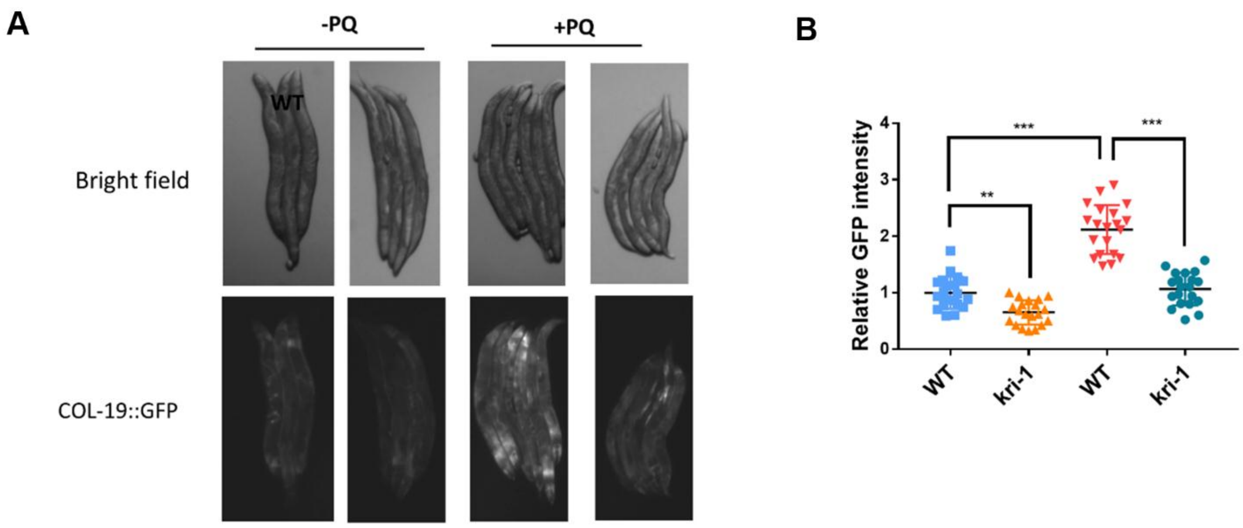

Supplementary Figure 1. COL-19::GFP expression at day-4 of adulthood. (**A**) Collagen fusion protein COL-19::GFP was increased by paraquat in a KRI-1 dependent manner. Wild type (WT) and *kri-1(ok1251)* mutant *C. elegans* expressing COL-19::GFP fusion protein were treated with 75 μM paraquat (PQ) from L1 to day-4 adulthood. Animals were imaged with fluorescence microscope. Shown are representative images from 2 experiments. (**B**) Quantification of GFP intensity in individual worms. 20 worms were selected from 2 independent experiments and quantified through ImageJ software. Data were normalized to the average of WT non-treated control. Error bars show the standard deviation of 20 worms. P values were obtained from two tailed, unpaired student's t-test (**, P<0.01, ***, P<0.001).

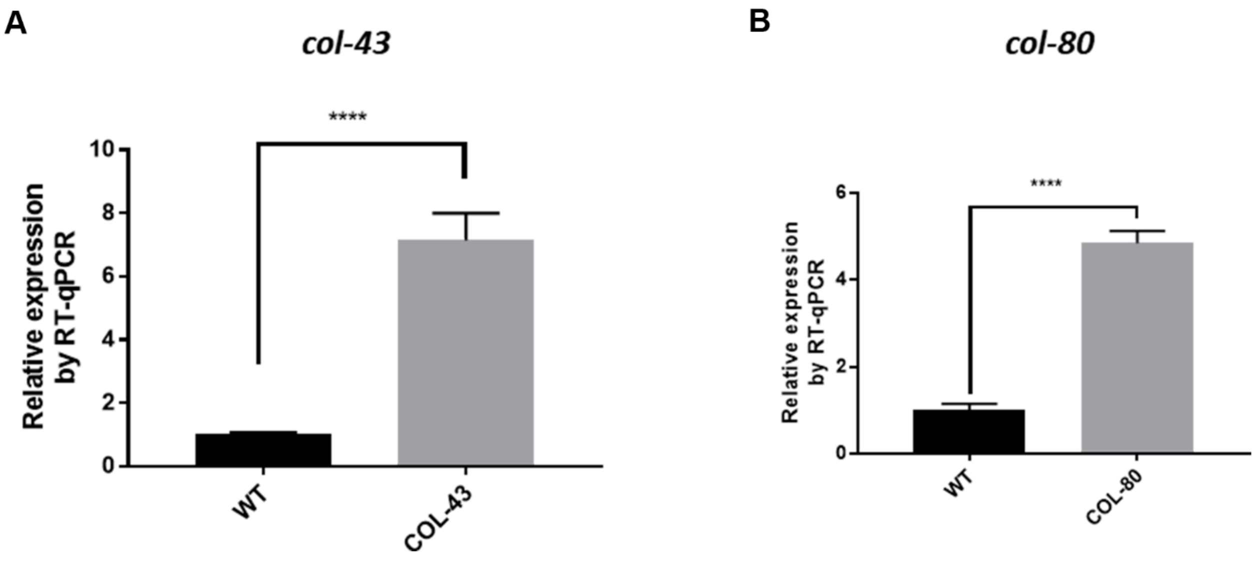

Supplementary Figure 2. Confirmation of COL-43 and COL-80 overexpression in transgenic lines. N2 wild-type (WT) *C. elegans* and strains expressing COL-43 (**A**) or COL-80 (**B**) were synchronized at L1 stage by hatching eggs in M9 buffer. Synchronized L1 animals were raised on standard NGM agar plate seeded with OP-50 from L1 to day-1 adulthood. Total RNA from day-1 adult worms was prepared for RT-qPCR analysis with primers for *col-43* and *col-80*. Data are the relative expression of *col-43* and *col-80* normalized to control RNAi and no drug control. Primers for actin gene was used as internal control. Error bars show the standard deviation of 4 replicates. P values were obtained from two tailed, unpaired student's t-test (****, P<0.00001).

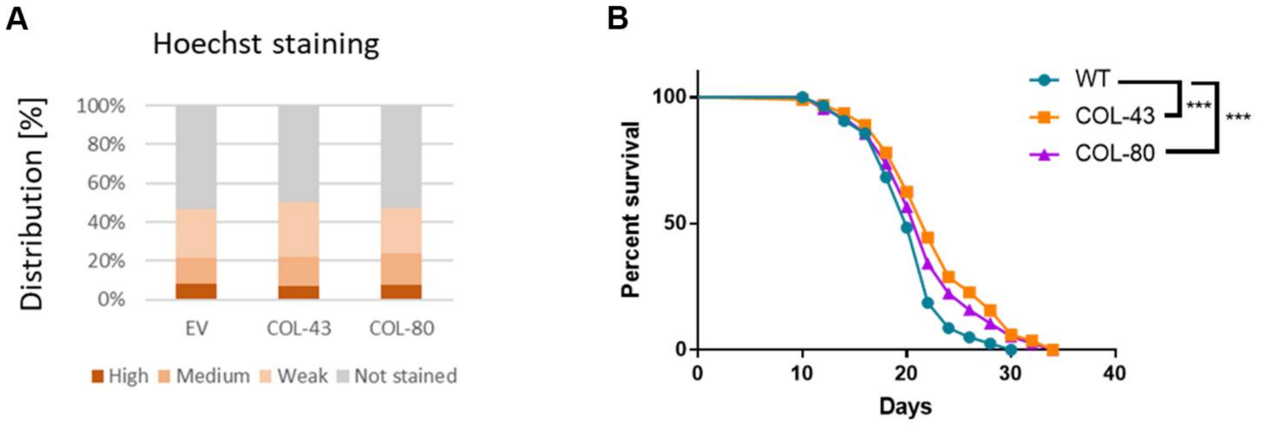

Supplementary Figure 3. (**A**) Collagen overexpression does not affect the Hoechst permeability through cuticle. Day-1 adult worms were incubated in 1 μg ml−1 Hoechst 33342 for 15 min and washed with M9 buffer. Stained nuclei in the tail area were examined under microscope. Shown are % of animals with high (>10 nuclei stained), medium (6-10 nuclei stained), low (1-5 nuclei stained) or no Hoechst staining. (**B**) Collagen overexpression extends lifespan of Wild-type *C. elegans*. Age-matched worms expressing COL-43 or COL-80 and wild-type (WT) controls were cultured on NGM medium at 20 °C from L1 to L4. 50 μM FUDR (5-Fluoro-2′-deoxyuridine) was added to inhibit reproduction. Worms may be transferred to new plates as required. Experiments were carried out in 2 biological repeats and data were pooled and shown in Kaplan Meier survival curve. Comparison between control and overexpressors were statistically analyzed by log-rank test in Prism software.
