# Supplementary material for: Modeling paraquat-induced lung fibrosis in C. elegans reveals KRIT1 as a key regulator of collagen gene transcription
Source: Aging (Albany NY). 2021 Jan 20;13(3):4452–67. doi: 10.18632/aging.202406 (PMC7906160; doi:10.18632/aging.202406)
Supplement: Supplementary Tables [file aging-13-202406-s003.pdf]

SUPPLEMENTARY TABLES

Supplementary Table 1. siRNA information.

siRNA target	Sequence	Source	Ref
NRF2	5’-UCCCGUUUGUAGAUGACAA-3<br>5’-UUGUCAUCUACAAACGGGA-3’	Shanghai GenePharma	[1]
KEAP1	5′-GGCCUUUGGCAUCAUGAACTT-3′<br>5′-GUUCAUGAUGCCAAAGGCCTG-3′	Shanghai GenePharma	[2]
KRIT1	Silencer Validated siRNA pools (siRNA ID 15655)	ThermoFisher Scientific AM51331	[3]

Supplementary Table 2. RT-qPCR primer sets information.

Gene	Primer sequence	Source
col-43	5′-CTTATTCTTTGAAATTTATTTTGC-3′<br>5′-AGTCTTCATGAAGTTGACTT-3′	Custom, Shanghai GenePharma
col-80	5′- GTAAGTACCATAAAAATACTTTG -3′<br>5′-GCAGAATCATCATGATTAAC-3′	Custom, Shanghai GenePharma
col-139	5′-AAAGAGCTTGCTCAATGCA-3′<br>5′-GATACTTTTTTCAGATTTCAGATC-3′	Custom, Shanghai GenePharma
act-1	5′-ACGACGAGTCCGGCCCATCC-3′<br>5′-GAAAGCTGGTGGTGACGATGGTT-3′	Reference [4]
COL27A1	5′- ggccttatggaaatccaggt-3′<br>5′- gcaagcccatgtcacctt-3′	Roche Probe Finder
COL28A1	5′-tctgcatattccatgagagtga-3′<br>5′-aggagggaacaaagcaagaag-3′	Roche Probe Finder
GAPDH	5′-AATCCCATCACCATCTTCCA-3′<br>5′-TGGACTCCACGACGTACTCA-3′	Reference [5]
